# Evaluation of the effects of health impact assessment practice at the local level in Monteregie

**DOI:** 10.1186/s12961-016-0076-5

**Published:** 2016-01-27

**Authors:** Kareen Nour, Sarah Dutilly-Simard, Astrid Brousselle, Pernelle Smits, Jean-Marie Buregeya, Julie Loslier, Jean-Louis Denis

**Affiliations:** Monteregie Regional Department of Public Health, Longueuil, QC J4K 2 M3 Canada; Faculty of Medicine and Health Sciences, Université de Sherbrooke-Campus Longueuil, Longueuil, QC J4K 0A8 Canada; Charles-Le Moyne Hospital Research Centre, Greenfield Park, QC J4K 0A8 Canada; École Nationale d’Administration Publique, Montréal, QC H2T 3E5 Canada

**Keywords:** Cities, Health impact assessment, Public health, Public policy

## Abstract

**Background:**

In Quebec (Canada), the Monteregie Regional Public Health Department has chosen to use health impact assessment (HIA) to support municipalities through a knowledge exchange and collaborative process in order to positively influence decision-making regarding local policies and projects. The value of HIA is becoming increasingly recognized by municipalities interested in planning and managing their cities with an eco-systemic perspective. However, the knowledge and tools which support the use of the HIA at regional and local levels are still missing.

**Methods:**

The general objective is to evaluate the impact the collaborative HIA process used in Monteregie has had on the formulation, adoption and implementation of policies and projects favourable to health. The methodology is based on Mayne’s CA design, which allows the identification of factors which contribute to a change process. It is described as one of the best approaches to reduce uncertainty regarding the observed results and the contribution of a program. All of the HIA processes realised between January 2013 and January 2016 in Monteregie will be studied following a case study strategy. Study populations include regional and local public health professionals, municipal officers and community members implicated in these HIAs. Various qualitative and quantitative methods will be used, including examination of documentation, observations on the city grounds, and individual or group interviews. A model of change will be constructed for each HIA process and will present the logical pathway which leads to the observed results, alternative explanations and hypothesises as to why these results were obtained, and contextual factors that could have influenced them. This model will allow the production of a refined contribution story for each HIA. A convergence and divergence analysis will be completed in order to identify differences or similitudes between the different HIAs studied.

**Discussion:**

In addition to contributing to the production of knowledge in relation to the collaborative model of HIA, this research project will allow other regional and local public health actors and municipalities of Quebec or other decision-making and political bodies to understand the usefulness of this approach for the improvement of population health and well-being.

## Background

### Health impact assessment (HIA) in the province of Québec, Canada

HIA is a means of prospectively assessing the health impacts on social and environmental determinants prior to implementation of intended policies, plans or projects. Knowing that public policies of different sectors (e.g. transport, education, housing) can affect the health of the population, HIA aims to inform decision-makers in their choice of alternatives to maximize positive and mitigate negative health impacts or actively promote health [1-3]. HIA has shown positive effects on intersectoral collaboration and strategic planning considering the health and wellbeing of the population [4,5].

The HIA practice in the province of Quebec, Canada, is used to ensure the application of article 54 of the Quebec Public Health Law, whereby all ministries are responsible to consult the Ministry of Health and to take into account the potential health impacts which can result from their policies and projects (legislation and regulation projects) [6-8]. Following international tendencies, HIA have been used primarily at the national level in Quebec, where they have informed national policies and inter-ministerial mechanisms [9]. Knowledge and tools have thus been developed by the Ministry of Health and Social Services to reply to this level of intervention and knowledge translation [6].

The usefulness of the HIA practice to act on health determinants and to promote healthy public policies is not limited to the national level. Its implementation at regional and local levels seems promising [4,10]. In 2011, the Monteregie public health department (PHD) chose to use HIA at a local level (for municipal projects, plans or policies).

### Research opportunity: HIA projects undertaken at a local level

Cities are becoming more aware and interested in planning and managing in an eco-systemic perspective, which can be seen by the increasing number of municipalities participating in the Healthy Cities movement [11,12]. The value of HIA is becoming increasingly recognized by municipalities interested in planning and managing their cities with an eco-systemic perspective [2,13]. In addition, the development of healthy public policy is increasingly expected by citizens and governments. In the province of Quebec, current and future orientations for government policy based upon sustainable development, land use and active transport are some examples. However, the knowledge and tools to support the use of the HIA at regional and local levels to inform the impact of municipal and territorial policy and projects on health are still missing.

One of the few comprehensive studies concerning the impact of the HIA processes at the local level demonstrated direct improvements, after an HIA process, in the planning and development of public health policies [14]. Indirectly, HIA encouraged policymakers to consider the unintended impacts of their decisions on the health of their citizens, facilitated intersectoral collaboration within local government, provided a systematic mechanism for evidence-based planning, encouraged ownership of municipal decisions by the community and made more transparent discussions in the decision-making of local government authorities.

Results from more recent international studies show similar results. They have also shown how HIA influenced decision-making in the development of local projects or policy, specifically in relation to the integration recommendations from the HIA and with improvement of intersectoral collaboration [4,5,15,16]. More specifically, in New Zealand, one study revealed positive direct effects from the HIA on the development of the urban development plan, wherein nearly two-thirds of the recommendations were included in the final plan [4]. Another study concluded that the HIA positively influenced several plans and local projects by facilitating the inclusion of considerations in relation to health. Impacts were found to be higher the earlier the initiation of the HIA in the elaboration of a policy or plan [15]. According to this research, in order to facilitate the integration of health in local projects, public health authorities must act in partnership with local planners by involving them very early in the HIA. Three studies highlighted that HIAs improve relations between public health authorities and local governments and, in some cases, with the community [4,5,15].

### The HIA practice in Monteregie, Quebec: study setting

The Canadian province of Quebec has a population of close to 8 million. Universal access to health and social services is afforded to the population through a revenue taxation system. Health and social services are composed of three forms of governance, including the national Ministry of Health and Social Services (MSSS), the local Integrated Health and Social Services Centres (CISSS) and the Integrated University Health and Social Services Centres.

In Monteregie, one of the 16 administrative regions in the Québec province, three CISSS (Monteregie-Centre, Monteregie-Est, Monteregie-Ouest) share the responsibility of serving a population estimated to be 1.4 million. The CISSSs assume the responsibility of the health and wellbeing of their population by working with a group of actors, which together form a territorial network of services and ensure the organization of the entire continuum of services within a framework of several missions (hospital centre missions, local community health centres, shelters, long-term care and rehabilitation).

The public health directorate of Monteregie is under the responsibility of the CISSS of Monteregie-Centre, but assumes the public health services for the entire Monteregie region. A regional public health action plan is compiled according to the directives of the national public health programme, in accordance with regional particularities and the needs of the local population. In Monteregie, the HIA was included in the 2011 regional public health action plan, despite the absence of this priority by the MSSS at the time. The HIA is thus a novel practice for Québec, and is implemented on a voluntary basis with the public health directorate in Monteregie. Notably, as part of the 2015–2025 national public health programme, the MSSS adopted this approach in its new practices.

The responsibility for the HIA in Monteregie is assumed by a professional who works full time as a knowledge broker at the public health directorate, collaborating across the three CISSS territories with front line health workers. These front line health workers, health promotion agents or community organizers establish links with municipalities in the territories as part of their work. Monteregie extends across a territory of 10,000 km^2^, with 177 municipalities in urban, semi-rural and rural regions. Monteregie equally includes regional county municipalities that administrate groups of several municipalities.

In Monteregie, HIA supports municipal decision-makers through a collaborative process. Specifically, HIA is based on a process of knowledge exchange between actors with general knowledge and expertise regarding health issues (public health professionals from the PHD) and those with local context-bound knowledge of health issues specific to a particular municipality (first line public health professionals, municipal actors and citizens). The co-construction of knowledge occurs as a result of this collaborative process. The aim of the HIA process is thus to render this knowledge accessible and relevant to municipal decision-makers.

A seven step approach, inspired by WHO’s five step approach [17], is used in Monteregie. The five steps under the WHO guideline consist of screening, scoping, analysis, recommendations and evaluation. The screening step aims to make a quick read of the policy or project (e.g. projects residential, plans) to identify the factors that can influence health. The framing step determines the extent of the analysis and the planning of the subsequent steps. The analysis is performed by a literature review, expert consultations from various teams of the PHD (e.g. Environment), and analysis of data and field observations; a report is then written, presenting an analysis of the potential impacts of the policy or project on health. This analysis is also represented by a schematisation of the change model, which illustrates the links between health determinants and the various elements of the policy or project. The report presents a series of recommendations in relation with this analysis. Still in relation with the health determinants, the recommendations can refer to socio-cultural (e.g. beliefs, values), economic (e.g. low cost activities), physical (e.g. changing or adding a bicycle path) or political (e.g. measurement of inclusion of citizens in decisions) dimensions [18]. Evaluation refers to presentation and discussion of the process with the actors involved to measure its overall appreciation.

The HIA implemented in Monteregie is different from the national and international models in many ways:

1. It involves a triad of actors: public health professionals (regional and local), municipal actors, and sometimes citizens (depending of the project’s nature).
2. It relies on a systematic seven-step collaborative process.
3. It provides to each participating municipality a report consisting of an assessment and recommendations.
4. It uses a process based upon co-construction of knowledge.
5. It is undertaken in a political context which, on the one hand, mandates public health to intervene on public health matters and, on the other hand, makes no such legal mandates for municipalities.

### Study objectives

In 2015, to better support municipal and local actors in the HIA process and to advance practice-based research, the PHD, Ministry of Health and Social Welfare, University of Sherbrooke, Charles-Le Moyne Hospital Research Centre and École nationale d’administration publique submitted a research proposal and obtained a Partnerships for Health System Improvement (PHSI) grant from the Canadian Institutes of Health Research (CIHR) and the Fonds de recherche – Santé Québec (FRSQ).

The research project aims to conduct an impact assessment according to Quigley’s typology [19] of the HIA process carried out in Monteregie. The general objective is to evaluate the impact of the collaborative HIA process on the formulation, adoption and implementation of policies and projects favourable to health.

The specific research questions are:

1. How is the knowledge produced and shared during the HIA process used by municipal decision-makers regarding the formulation, adoption and implementation of projects or public policies favourable to health?
2. What contextual factors (political or economic) and personal (commitment, values and beliefs) influenced this decision-making?
3. To what extent are impacts observed on decision-making attributable to the HIA process?

### Theoretical framework of change: Advocacy Coalition Framework

The Advocacy Coalition Framework [20] was selected to guide our understanding of the formulation, adoption and implementation of municipal policies or projects favourable to health following the HIA process. It has been used in several studies on the HIA process and public policies favourable to health [21]. This framework describes the subsystems of actors that influence public policies in different areas. The actors from these subsystems may be inside or outside a government body (citizens, journalists, experts, *etc*.). The framework also considers the cognitive and normative dimensions of actors who are grouped according to common beliefs and values. Sabatier and Jenkins-Smith [20] argue that changes in values or beliefs are related to the confrontation of actors to new or different belief and values systems. The development and adoption of public policies favourable to health are described by three axes:

1. The decision-making process, conditioned by institutional, political, normative and cognitive dimensions (knowledge and values of actors involved: beliefs, resources, and strategies).
2. The prospective evaluation of the public policy process, refering to the evaluation of the potential effects of policies on some determinants of health.
3. The transfer and appropriation processes, meaning the use of knowledge. Information and scientific knowledge used by professionals in their practices come from various sources. To make the sharing of knowledge the most effective it requires the involvement of all partners.

The external environment, including the decisions taken by other subsystems (e.g. government, regional elected group, regional county municipalities), as well as public opinion, are factors influencing the formulation and adoption of projects or public policy.

The Advocacy Coalition Framework therefore demonstrates that many factors can influence the development and adoption of public policies favourable to health. Since the HIA process aims to advance municipal stakeholders’ interests and capacities to consider the health impact of a policy or project, our research aims to identify the contribution the HIA process makes when the policy or the project becomes implemented. This theoretical framework comprehensively identifies the factors that could influence the observed results and further highlights the contribution and the role the environmental context plays in the decision-making process.

**Fig. 1:**
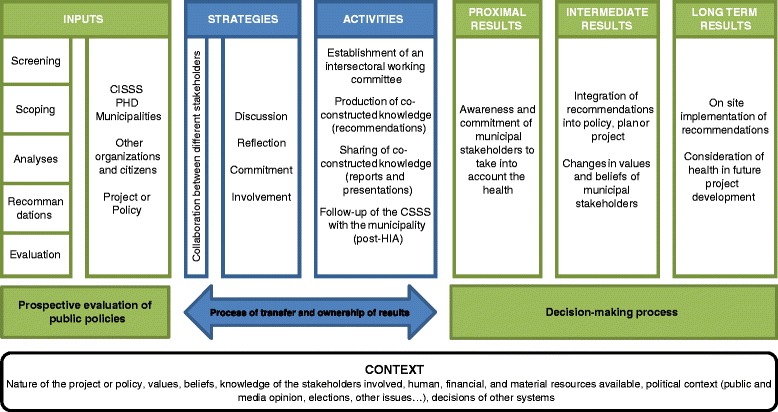
Logic model of the HIA process in Monteregie

The three axes of the Advocacy Coalition Framework were used to develop the logic model of the HIA process undertaken in Monteregie (Fig. 1). The HIA process allows a prospective evaluation of projects or public policy while the strategies and activities carried out, considering their collaborative nature, allow a transfer and ownership of knowledge by the actors involved. To explore the role of the context and the HIA process in decision-making, the Advocacy Coalition Framework recommends the use of a flexible and comprehensive evaluative framework.

## Methods

### Research design

Improving health in cities means intervening in a complex setting and HIA can be considered a complex intervention [11,22]. Traditional experimental and quasi-experimental design cannot be applied for evaluating the effectiveness of such complex interventions. Our research design is inspired by the CA approach [23], which aims to identify impacts from public policies, particularly when the causal links are not strong enough to allow the use of more traditional research designs [24,25].

CA proposes an approach that permits the identification of factors which contribute to a change process [23]. It is described as one of the best approaches to reduce uncertainty regarding the contribution of a program to the observed results [26], and its framework takes into account the complexity of multiple influences. It therefore uses different types of data collection methods in order to understand how a program works, why it works and in what contexts [27]. Moreover, although this approach does not allow to measure causality, it adequately documents the factors which contribute to program success [27].

The research design was inspired by the six steps of the CA, which we slightly adapted to better suit our specific needs:

- Step 1. Develop a model representing the anticipated chain of results (the program theory) – the chain of results (proximal, intermediate, and long term), and the external or contextual factors that may influence them, are presented clearly in this model.
- Step 2. Assess the existing evidence regarding the results – the risks and assumptions that may influence the chain of results are identified. Evidence on the strength of influence of these risks and assumptions on the chain of results is gathered.
- Step 3. Assess alternative explanations – plausible alternative explanations influencing the chain of results are identified and evidences are seeked to assess the strength of their influence. When its influence is considered to be low, an alternative explanation is ruled out.
- Step 4. Assemble the contribution story – this describes the program context, the planned and actual accomplishments, the lessons learned, the approach implemented to ensure the quality of information, the alternative explanations contributing to the outcomes, and a clarification of why they had no, or limited, influence.
- Step 5. Seek additional evidence – when the alternative explanations cannot be ruled out or when the program (e.g. HIA) cannot be demonstrated as being a potential contributor to the observed results, the model of change is reviewed and/or additional information is sought.
- Step 6. Revise and strengthen the contribution story – when further evidence fails to explain the contribution story, two options are faced: an additional analysis is required (revisit step 4) or the program cannot be considered to have contributed to the observed results.

### Study population and sample

The study populations are the PHD and first line public health professionals and municipal actors who participated in a HIA process in Monteregie between January 2013 and January 2016. Ten HIA were completed during this period and will all be evaluated. Each HIA must be completed (including a preliminary report presented to the municipality) at least 6 months prior to the first data collection. The realisation of this study depends upon the voluntary participation of the different stakeholders. Finally, each HIA process will be evaluated following a case study strategy. The case study is particularly appropriate when we seek to better understand the implementation process of an intervention in relation with the observed results [28].

### Data collection and analysis

Various qualitative and quantitative research methods will be used. Collection methods are derived from the steps of the CA [23]. For each of the steps planned by this type of design, the variables studied, methods of data collection and analysis are presented in Table 1.

**Table 1:** Variables to be studied, data collection and analysis methods for each step of the CA

Contribution analysis (CA)	Variables	Data collection methods	Analysis
Step 1. Develop a model representing the anticipated chain of results (program theory)	•Anticipated or observed results ^a^ of the HIA	•Informal individual interview with the PHD knowledge broker	Information will be synthetized and presented in the form of a logic model illustrating chain of results, influencing factors and alternative explanations
	•Contextual elements which could have positively or negatively influenced these results	•Examination of documentation (e.g. Political reports, media documents, etc.)	
Step 2. Assess the existing evidence regarding the results	•History of the city’s involvement in an HIA	•Individual interview with the local public health professional who participated in the HIA	The verbatim of the interviews will be transcribed and analyzed; data will be coded using the NVivo (QSR). The validation will be conducted by a double coding technique. The inter-coder technique will be used by the research coordinator and a researcher, who will undertake an independent and parallel encoding for the first verbatim and compare their results. In case of disagreement, they will clarify their differences, refine the codes and will resume encoding. This process will be repeated until a 90% inter-coder reliability is achieved [32].
	•HIA process: actors involved and collaboration process	•Individual interview with the municipal authority of the HIA (could be the mayor, city councillor or a member of municipal staff such as an urbanism director)	
	•Perceived contribution of the HIA process on the results (depending on the model of change developed in step 1)		
	•The elements of the context which may have influenced the results		
Step 3. Assess alternative explanations	•Perceived contribution of the HIA process on the results (depending on the model of change developed in steps 1 and 2)	•Focus group or individual interviews with other local actors who participated in the HIA	
Step 4. Assemble the contribution story	This step will allow the research coordinator to interpret and synthesize the results from steps 1 to 3. The coordinator will produce an explanatory document which syntheses the story. References to the specific data sources to support statements will be used. This document will be revised by a working group composed of the principal researcher, the research coordinator and the knowledge broker of the PHD.		
Step 5. Seek additional evidence	According to the robustness of the story reached as of step 4, additional evidence will be collected if necessary, concerning the role of different factors which may have influenced the results. This could imply the revision of municipal meeting report or statements, examination of other documentation produced by the municipality, or seeking additional information by realising a second individual interview with the municipal authority of the HIA.		
Step 6. Revise and strengthen the contribution story	The research coordinator will interpret and synthetize results during step 6. The coordinator will produce a refined summary of the contribution story, based on the information obtained through steps 1 to 5. This summary will be validated using the same process outlined in step 4. In addition, the steering committee will be asked to comment on this summary. A working group will discuss their overall experience with each of the HIA in general and the impact the HIA had on policy and practice within each of the municipalities. Their experiences and interpretations will be compared to the results obtained from the data analysis and the contribution stories.		

^a^Anticipated results of an HIA include sensibilisation of the actors to consider health, integration of the recommendations in the project, plan or policy, change in values or beliefs of the actors, changes in the actual project following the recommendations, and integration of health considerations in other municipal projects following the HIA.

Following steps 1 to 6, the contribution story (each case study should have one contribution story) will be assembled by the research coordinator, who will complete an initial analysis of the results obtained. A convergence and divergence analysis will be completed in order to identify differences or similitudes between the different HIAs studied. This analysis will also be related to the logic model of the HIA process (Fig. 1), and Sabatier and Jeankins’ model [20], which will allow an exploration of the role the HIA process played in the decision-making process; this analysis will be validated by the research working group.

### Study validity

1. Construct validity: ensured by the creation of a detailed logic model specifying the studied variables [29].
2. Intern validity: ensured by the utilization of an analytic framework, a rigorous organization of the collected data, and a systematic codification technique. It is also strenghtened by the triangulation of data, the analysis of different perspectives and the choice of different sources of data [30].
3. External validity: strenghtened by the multiple case study method [29] and the variability of the cases (e.g. type of project under HIA, municipality size, urban or rural). The detailed description of each HIA studied will allow judgments to be made on the pertinence or applicability of the results to other municipal contexts.
4. Fidelity: strengthened by the detailed description of each step in the research project for each case study. The procedure used for each of the research activities will also be described.

### Trial status

When our research proposal was submitted, five HIA processes had been completed in Monteregie. These HIA include a housing development project, a revitalization plan, a social policy, an urban design plan and the plan of an institutional pole [31]. Municipalities in Monteregie vary in size and thus the implementation contexts of the HIA varied from small rural areas with close to 1,000 citizens to larger urban centres with more than 50,000 citizens. The health determinants addressed in these five HIAs are related to the natural environment (air, water, soil), the built environment (transport infrastructure, noise), activities and services (housing and leisure), and the community (social capital).

As of January 2016, data collection (steps 1 to 3) has been started or completed for six HIA cases: two Strategic Development Plans, one local policy and action plan for elders, and three residential sectors to be built. Data collection will continue for two years with four additionnal HIAs.

## Discussion

HIA practice at a local level has been the focus of a few international studies. To our knowledge, this is the first study exploring the effects of HIAs realised in collaboration with cities in Quebec. The use of CA and the methodology developed in this research project are expected to reduce uncertainty regarding the influence of HIA interventions on decision-making processes at a local level. Although this approach does not allow to measure causality, it will certainly allow adequate documentation of the factors leading to its success [27] by taking into account the complexity of multiple factors of influence. Therefore, in addition to contributing to the production of knowledge on the collaborative model of HIA, this research project will allow other regional and local public health actors and municipalities of Quebec, as well as other decision-making and political bodies, to understand the usefulness of this approach for the improvement of health and well-being of the population.

## Abbreviations

HIA: Health impact assessment
PHD: Public health department
CA: Contribution analysis
